# 

**Published:** 1882-03

**Authors:** 


### BOOKS AND PAMPHLETS RECEIVED.

Diseases of Old Age. Charcot. Medical Library, Wm. Wood & Co., 1881.

Practical Normal Histology. By T. Mitchell Prodden. New York, Putnam's Sons.

Diseases of the Eye. By Henry D. Noyes. New York, Wm. Wood & Co. Library, 1881.

Organic Materia Medica. John M. Moich, Ph.D. Henry C. Lea's Son & Co., Philadelphia, 1882.

Nervous Diseases. By A. McL. Hamilton. H. C. Lea's Son & Co. Philadelphia.

Illustrations of Dissections. Ellis. Wm. Wood & Co. Medical Library 1882.

Diseases of Women. Edis. London. H. C. Lea's Son & Co., Philadelphia.

International Encyclopædia of Surgery. Vol. I. Wm. Wood & Co., New York. W. T. Keener.

Sympathetic Diseases of the Eye. L. Manthuer. Wm. Wood & Co.

A Manual of Ophthalmic Practice. Henry S. Schell, Philadelphia. D. G. Brinton.

Essentials of Practical Medicine. Hartshorne. H. C. Lea's Son & Co. Philadelphia. Jansen, McClurg & Co.

SOCIETY MEETINGS.

Chicago Medical Society—Mondays, March 6 and 20.
Chicago Pathological Society—Monday, March 13.
Biological Society—Wednesday, March 4.

CLINICS.

MONDAY.

Eye and Ear Infirmary—1.15 p. m., Otological, by Prof. Jones; 2.15 p. m., Ophthalmological, by Prof. Hotz.

Mercy Hospital—2 p. m., Medical, Profs. Hollister and Quine.

Woman's Medical College—2 p. m., Dermatological and Venereal, by Prof. Maynard; 3 p. m., Diseases of the Chest, Prof. Ingals.

TUESDAY.

Rush Medical College—3 p. m., Dermatological and Venereal, by Prof. Hyde.

Cook County Hospital—2 to 4 p. m., Medical and Surgical Clinics.

Mercy Hospital—2 p. m., Surgical Clinic, by Prof. Andrews.

WEDNESDAY.

Chicago Medical College—2 p. m., Eye and Ear, by Prof. Jones.

Rush Medical College—2 p. m., Medical, by Dr. Bridge; 3 p. m., Ophthalmological and Otological, by Prof. Holmes; 3:30 to 4:30 p. m., Diseases of the Chest, by Dr. E. Fletcher Ingals.

Eye and Ear Infirmary—2.30 p. m., Dr. E. J. Gardiner.

THURSDAY.

Chicago Medical College—2 p. m., Gynæcological, Prof. Jenks.

Rush Medical College—2 p. m., Diseases of Children, by Dr. Knox; 3 p. m., Diseases of the Nervous System, by Prof. Lyman.

Eye and Ear Infirmary—2 p. m., Ophthalmological, by Dr. Hotz.

Woman's Medical College—3 p. m., Surgical, by Prof. Owens.

FRIDAY.

Cook County Hospital—2 to 4 p. m., Medical and Surgical Clinics.

Mercy Hospital—2 p. m., Medical, by Prof. Davis.

SATURDAY.

Rush Medical College—2 p. m., Surgical, by Prof. Gunn; 3 p. m., Orthopœdic, by Prof. Owens.

Mercy Hospital—2 p. m., Surgical Clinic, by Prof. Andrews.

Chicago Medical College—3 p. m., Neurological, Prof. Jewell.

Woman's Medical College—11 a. m., Ophthalmological, by Prof. Montgomery; 2 p. m., Gynæcological, by Prof. Fitch.

Daily Clinics, from 2 to 4 p. m., at the Central Free Dispensary, and at the South Side Dispensary.

